# Nutritional intervention trial of yeast mannans derived from baker's yeast enriches *Bacteroides* and *Parabacteroides* with concurrent reduction of *Klebsiella pneumoniae*

**DOI:** 10.3389/fmicb.2026.1958679

**Published:** 2026-10-30

**Authors:** Melissa L. Moreno, Diana H. Taft, Thomas A. Tompkins, Wendy J. Dahl

**Affiliations:** 1 Food Science and Human Nutrition Department, University of Florida, Institute of Food and Agricultural Sciences (IFAS), Gainesville, FL, United States; 2 Lallemand Bio Ingredients, Montreal, QC, Canada

**Keywords:** *Bacteroides*, *Klebsiella pneumoniae*, metabolomics, microbiome, *Parabacteroides*, whole metagenome sequencing, yeast mannans

## Abstract

**Introduction:**

Supplementation with a yeast cell wall fraction rich in mannans is well tolerated in adults without gastrointestinal disease; however, limited research has examined its selective utilization by the gut microbiota and associated health benefits. The aim of this analysis was to investigate the specific effects of yeast mannans on the composition and metabolic function of the gut microbiota.

**Methods:**

Fecal samples from an open-label study of adults consuming 15 g of yeast mannans per day were profiled for fecal microbiota composition using whole metagenome sequencing, and fecal and urine metabolomics were analyzed by direct infusion and liquid chromatography-tandem mass spectrometry (DI-MS/MS and LC-MS/MS).

**Results:**

Differential abundance testing identified 46 species whose relative abundances increased with the yeast mannan intervention, primarily in the genera *Bacteroides* and *Parabacteroides*. *Thomasclavelia spiroformis* showed the largest fold increase. Four species decreased in relative abundance, with a greater-than-2-fold decrease in *Klebsiella pneumoniae*, with 45% of participants showing no detection following the yeast mannan intervention. Minor metabolomic changes were noted, including increased urinary excretion of *N*-acetylalanine, alanine, and caproic acid, and decreases in fecal concentrations of ether-linked phosphatidylcholines C36:4 and C40:1.

**Discussion:**

Yeast mannan supplementation selectively enriched several *Bacteroides* and *Parabacteroides*, saccharolytic commensals that may outcompete *Klebsiella pneumoniae*, an opportunistic pathobiont capable of causing extraintestinal infections.

## 1 Introduction

Prebiotics have been investigated for more than 30 years (Gibson and Roberfroid, 1995); however, identifying potential substrates that meet the criteria of being selectively utilized by gut microbiota and providing a measurable human health benefit (Gibson et al., 2017) without adverse gastrointestinal symptoms has remained somewhat elusive. Traditional prebiotics such as fructo-oligosaccharides (FOS) and oligofructose have been associated with gastrointestinal symptoms in healthy individuals (Mysonhimer and Holscher, 2022) and intolerance in patient populations (Chumpitazi et al., 2018), due to their rapid fermentation and propensity to induce bloating and flatulence (Mysonhimer and Holscher, 2022). For individuals with irritable bowel syndrome, avoiding these prebiotics is required when following the low FODMAP (Fermentable Oligosaccharides, Disaccharides, Monosaccharides, and Polyols) diet, which is effective in reducing gastrointestinal symptoms (van Lanen et al., 2021). Recent evidence suggests that a diet low in FOS and galactooligosaccharides (GOS) provides relief similar to that of the standard, extensively restrictive low-FODMAP diet (Jensen et al., 2025). Furthermore, the specificity of inulin-type fructans in modulating microbiota composition has been challenged by findings of broad effects on the gut ecosystem (Vandeputte et al., 2017). Specifically, regarding FOS supplementation, the literature generally reports increased abundance of *Bifidobacterium* spp. (Dou et al., 2022). However, FOS is widely added to medical foods, such as enteral nutrition formulations, and may not have the same effects on microbiota composition in patient populations (Majid et al., 2014). Potentially deleterious effects of FOS on the microbiome in critically ill patients include suppression of *Bifidobacterium* spp. and enhancement of Enterobacteriaceae opportunistic pathogens (Serbanescu et al., 2025).

In contrast, evidence suggests that dietary supplementation with yeast mannans derived from baker's yeast, offering potential health benefits (Wei, 2025), is well-tolerated (Tanihiro et al., 2023), which may in part be due to the slower and more selective breakdown (Cuskin et al., 2015) vs. highly soluble and rapidly fermentable oligosaccharides, typical of most prebiotics. Some members of the phylum Bacteroidota (formerly Bacteroidetes) use yeast mannans; a prime example is *Bacteroides thetaiotaomicron* (Cuskin et al., 2015), an abundant organism in the human microbiota (Xu et al., 2003). Findings from *in vitro* fecal fermentation using 16S rRNA gene sequencing demonstrate increased *Bacteroides thetaiotaomicron* in response to yeast mannans (Oba et al., 2020a,b), and *B. thetaiotaomicron* and *Bacteroides ovatus* were enhanced in response to yeast mannan supplementation *in vivo* (Tanihiro et al., 2020). Beyond compositional changes, yeast mannan supplementation lowered fecal concentrations of *p*-cresol, indole, and skatole (Tanihiro et al., 2020), long-established products of microbial metabolism of aromatic amino acids (Cummings and Macfarlane, 1991). These inflammatory compounds are known to contribute to uremia and toxicity in chronic kidney disease (Harlacher et al., 2022), adverse cardiovascular outcomes (Nemet et al., 2023), and clinical severity of neurodegenerative disease (Chen et al., 2023) and neurotoxicity (Ntranos et al., 2022). Our *ex vivo* fermentation data demonstrated that yeast mannans stimulate *Bacteroides faecis, Bacteroides ovatus, Parabacteroides merdae, Parabacteroides distasonis*, and also *Bifidobacterium* spp. across human donor samples (Moreno et al., 2025). Similarly, supplementation with yeast mannans increased fecal levels of *B. faecis, B. ovatus, P. merdae*, and *P. distasonis*, but not *B. thetaiotaomicron* in generally healthy adults (Moreno et al., 2025). Because this lack of *B. thetaiotaomicron* contrasts with findings from Tanihiro et al. (2020, 2023), the specific effects of yeast mannan on human gut microbiota composition warrant further exploration. To date, only 16S gene amplicon sequencing, with its limited taxonomic resolution, has been used to assess the effects of yeast mannans on the composition of the human gut (fecal) microbiota.

Prebiotics, commonly added to processed foods and beverages and included in nutritional supplements, are consumed not only by healthy individuals but also by those with acute and chronic diseases, and some with immune suppression; thus, a deeper understanding of the effects of established and novel prebiotics on the microbiota and its metabolic activity is needed. Therefore, we investigated the effects of yeast mannans on the composition and metabolic function of the gut (fecal) microbiota using whole metagenomic sequencing, taxonomic profiling, and metabolomic analysis. It was hypothesized that yeast mannan supplementation would selectively increase the relative abundance of *Bacteroides* and *Parabacteroides*.

## 2 Materials and methods

### 2.1 Study design

Fecal and urine samples from a 3-week open-label study (1-week baseline and 2-week intervention) of yeast mannan supplementation were assessed for effects on the fecal microbiome and fecal and urinary metabolomics. Details of the study design, including inclusion and exclusion criteria, and primary outcome reporting on gastrointestinal tolerance and stool frequency have been previously published (Moreno et al., 2025). In brief, participants (*n* = 20) consumed 15 g/d yeast mannans, a *Saccharomyces cerevisiae* fraction primarily containing mannooligosaccharides, mannoproteins, and residual beta-glucan (45% ± 5% dietary fiber; ≥25% protein; < 2.5% fat; ManOliSa^TM^, Lallemand Inc., Montreal, QC, Canada). Specifically, the product contains 25%−45% mannooligosaccharides as determined by High-Performance Liquid Chromatography (HPLC). The study was approved by the University of Florida Institutional Review Board 1 and registered at clinicaltrials.gov (NCT05939336). In brief, consenting adults (≥50 years) collected fecal samples (Fisherbrand™ Commode Specimen Collection System) and 24-h urine at the end of baseline and intervention periods; aliquots were frozen at −80 °C.

### 2.2 Whole metagenome sequencing

Whole metagenome sequencing was performed by AmpSeq (Gaithersburg, MD). Genomic DNA was extracted using the ZymoBIOMICS DNA Miniprep Kit (D4300) per the manufacturer's instructions, and DNA concentrations were quantified with a Qubit fluorometer to ensure accurate input for library construction. Libraries were prepared using the NEBNext Ultra II FS DNA Library Prep Kit for Illumina, following the manufacturer's recommendations for enzymatic fragmentation and adapter ligation. Library quality was assessed using multiple complementary approaches: concentrations were verified with both Qubit and qPCR, and fragment size distributions were evaluated using a Qsep automated fragment analyzer to confirm appropriate insert size profiles (approximately 300–700 bp) prior to sequencing. Sequencing was performed on the Element Biosciences AVITI platform using a 2 × 150 cycle flow cell.

### 2.3 Read cleaning and host sequence removal

Raw sequencing reads were processed to remove adapters, low-quality bases, and host (human) DNA sequences. Adapter and quality trimming were performed using Trim Galore (v0.6.10) with cutadapt (v4.5) using parameters –paired, –cores auto, –quality 20, and –length 50. These thresholds remove bases with Phred scores below 20 and discard reads shorter than 50 bp after trimming. Human reads were removed by aligning trimmed reads to the CHM13v2.0 human reference genome using Bowtie2 (v2.5.2) with the –very-sensitive preset. Only paired reads unmapped to the human genome were retained using samtools (v1.20) with the filters -f 12 -F 256. Processed reads were evaluated using FastQC (v0.12.1) and MultiQC (v1.15) to confirm read quality and completeness.

### 2.4 Taxonomic profiling

Taxonomic classification of cleaned paired reads was performed using Kraken2 (v2.1.3) (Wood et al., 2019) with the Genome Taxonomy Database (GTDB) release R226 bacterial and archaeal reference (Parks et al., 2022). Kraken2 was executed in paired mode with—use-names options, default k-mer length of 35. Abundance re-estimation was conducted using Bracken (v2.9) (Lu et al., 2017) with parameters -r 150 (read length) and -l S (species levels). Bracken redistributes Kraken2 read counts based on read length-specific k-mer distributions to improve abundance estimation accuracy. In addition to taxonomic profiling, beta-diversity analysis was performed using Bray-Curtis distance and visualized using non-metric multidimensional scaling (Supplementary Figure S1).

### 2.5 Data integration and filtering

Species-level Bracken tables were merged and imported into R (v4.3.2) using the phyloseq package (v1.44) (McMurdie and Holmes, 2013), together with sample metadata and GTDB-based taxonomy annotations. To reduce sparsity and minimize noise from low-abundance features, taxa were retained only if their within-sample relative abundance exceeded 0.0005 (0.05%) in at least 10% of samples. Relative abundances were computed solely to evaluate this filtering criterion; the filter was then applied to the original count table, and unnormalized Bracken counts were carried forward to differential abundance testing, as ANCOM-BC2 operates on counts and internally estimates sampling fractions to perform bias correction. These thresholds were selected to exclude rare taxa that contribute disproportionately to compositional variability in small datasets.

### 2.6 Differential abundance analysis

Differential abundance testing between baseline or intervention (yeast mannan) groups was performed on filtered, unnormalized species-level counts using ANCOM-BC2 (v2.4.0) (Lin and Peddada, 2024), a compositional method that applies bias correction and allows for random effects. A mixed-effects model was used, in which log-transformed taxon abundance was modeled as a function of Time, where Time (preintervention, postintervention) was included as a fixed effect and Subject was modeled as a random intercept to account for repeated measures. The following model parameters were used: struc_zero = TRUE, prv_cut = 0.1, pseudo = 0.5, pseudo_sens = TRUE, s0_perc = 0.1, p_adj_method = “BH,” and alpha = 0.05. Taxa with false discovery rate–adjusted q-values < 0.05 were considered significantly different between conditions.

### 2.7 Metabolomics analysis

As reported previously (Moreno et al., 2025) urine metabolomics were analyzed by direct infusion and liquid chromatography-tandem mass spectrometry (DI-MS/MS and LC-MS/MS), using positive and negative-mode ionization multiple reaction monitoring. The TMIC MEGA metabolomics kit protocol was followed to process urine samples, and mass spectra were collected using an AB Sciex QTrap MS (Sciex, Framingham, MA, USA). Raw data were processed with the LC-Autofit (https://dev-lcautofit.wishartlab.com/; last accessed January 6, 2025) to identify and quantify metabolites. Methodological details are available elsewhere (Zhang et al., 2024a). In brief, for the non-lipid metabolites, the LC-MS panel of the MEGA assay was used (Zhang et al., 2024a). Authentic standards were used for metabolite identification, with identification based on matching the retention time (RT) and multiple reaction monitoring (MRM) transitions of the authentic standards. For absolute quantification, isotopically labeled internal standards were used together with a seven-point calibration curve. The assay was performed in a 96-well-plate format. The first 14 wells were allocated to blanks, double blanks, quality control (QC) samples, and calibration standards. For lipid metabolites, the flow-injection panel of the MEGA assay was used. Because authentic standards and isotopically labeled internal standards are not available for every lipid species, a minimum of one authentic standard per lipid class was used for identification based on MRM transitions. In addition, at least one internal standard per lipid class was used for quantification.

### 2.8 Metabolomics data processing and structure harmonization

Raw fecal and urine metabolomics datasets were imported from multi-row TSV files containing instrument-specific headers and metadata. Metabolite identifiers were parsed from the first row, while analytical limits of detection (LOD) were extracted from the third row. Quantitative data were obtained beginning at row four. All measurements were converted to numeric format, with zero or negative intensities recoded as missing. Sample metadata files were imported separately and aligned to quantitative matrices. All preprocessing procedures were implemented in R using a custom script.

### 2.9 Sample classification and paired design enforcement

Samples were assigned to baseline or intervention groups based on naming conventions. Subject identifiers were extracted using pattern matching to enable longitudinal alignment. Only subjects with complete baseline–intervention measurements in both fecal and urine datasets were retained. Paired design matrices were constructed by ordering samples such that all baseline samples preceded corresponding intervention samples for each subject.

### 2.10 Missing value characterization and feature filtering

For each metabolite, we quantified the proportion of missing values separately in baseline and intervention samples. Missingness distributions were visualized using per-metabolite histograms, and per-sample missingness was summarized using barplots. Features were removed only if they exceeded 30% missingness in both biological conditions, thereby preserving metabolites reliably detected in at least one physiological state. This conservative filtering avoids systematic exclusion of condition-dependent signals while controlling for pervasive data sparsity.

### 2.11 Hierarchical missing value imputation

The remaining missing values were imputed using a hierarchical strategy designed to preserve biochemical interpretability at low abundance. When a metabolite had a valid LOD, missing values were imputed as one-half of the LOD. If no LOD was reported, missing values were replaced with one-half of the minimum observed non-missing value. For metabolites lacking any non-missing values, the median intensity across the dataset was used. This approach ensures biologically plausible imputation while avoiding artificial truncation of metabolite distributions.

### 2.12 Fecal normalization by dry weight

Fecal metabolite intensities were normalized to dry weight based on experimentally measured water content for each sample. For each specimen, the dry weight fraction was calculated as the proportion of the sample remaining after subtracting the measured water content. All metabolite intensities were then scaled by this dry weight fraction. This correction reduces variability introduced by differences in stool hydration across samples.

### 2.13 Urine creatinine normalization

Urine metabolites were normalized to creatinine concentration when available. All metabolite values were divided by creatinine intensity, and creatinine itself was removed prior to downstream analyses.

### 2.14 Log2 transformation, generation of final analytical matrices, and principal component analysis

To stabilize variance and address right-skewed metabolite distributions, normalized intensities were log2-transformed. Paired baseline–intervention matrices were generated for fecal and urine datasets with matched subject ordering. A set of metabolites present in both matrices after quality control was identified for cross-matrix integrative analyses. To characterize global shifts in fecal and urine metabolomes between baseline and intervention, unsupervised principal component analysis (PCA) was performed on the paired datasets (see Supplementary material).

### 2.15 MaAsLin3 analysis

Associations between metabolite abundances and intervention status were assessed using MaAsLin3 (Nickols et al., 2026), which fits multivariable linear mixed-effects models for each metabolite. log2-transformed metabolite intensities were analyzed without additional normalization, and models included Condition (Baseline vs. Intervention) as a fixed effect and Subject as a random intercept to account for the paired study design. Minimal filtering was applied (min_abundance = −100; min_prevalence = 0) to retain all detected metabolites. Multiple testing correction was performed using the Benjamini–Hochberg false discovery rate, and metabolites with *q*-values < 0.2 were considered significant.

### 2.16 Pairwise sparse Partial Least Squares (sPLS) integration of metabolome and microbiome

To identify coordinated cross-omic relationships between the microbiome and host metabolites, pairwise sparse Partial Least Squares (sPLS) integration was performed across all four combinations of metabolomic and metagenomic datasets: fecal metabolome and fecal genus abundances, fecal metabolome and fecal species abundances, urine metabolome and fecal genus abundances, and urine metabolome and fecal species abundances. All analyses were implemented using mixOmics with the paired design incorporated via a multilevel sPLS framework to remove between-subject variation and retain within-subject structure. Metabolite matrices were the centered log-ratio–transformed intervention-response matrices generated from multilevel preprocessing; microbiome matrices were CLR-transformed genus- or species-level abundance tables filtered for minimum prevalence (>20%) and abundance (>0.0001). Metabolite and microbiome samples were matched by subject and condition using deterministic ID parsing. For each integration, the sPLS model was tuned using repeated five-fold M-fold cross-validation (five repeats), selecting the optimal number of metabolites (keepX) and microbial taxa (keepY) based on minimal mean absolute error. Two latent components were retained to capture the dominant cross-omic axes. From the fitted models, the top-contributing features (metabolites and genera/species) for Component 1 were extracted and computed metabolite–taxon correlation matrices. Strong cross-omic relationships were defined as |r| > 0.6, consistent with prior multi-omics literature. Network and heatmap visualizations were generated to summarize the leading cross-omic modules. To identify microbe–metabolite relationships that may underlie host metabolic responses to the intervention, pairwise sparse Partial Least Squares (sPLS) correlation analysis was performed between microbial abundances (genus-level and species-level) and targeted metabolomics profiles.

## 3 Results

### 3.1 Robust taxonomic alterations following yeast mannan supplementation

Differential abundance testing identified 50 species whose relative abundances differed between baseline and the yeast mannan intervention at *q* < 0.05 (Figure 1), with 23 passing the sensitivity analysis (Figure 2). Forty-six species increased in relative abundance, and four decreased. The dominant theme of the taxonomic changes was the expansion of the relative abundance of Bacteroidota, with 34 of the increased species belonging to the genera *Bacteroides* or *Parabacteroides*. The single species with the largest fold-change was *Thomasclavelia spiroformis* (formerly *Clostridium spiroforme*) (Kaneuchi et al., 1979; Lawson et al., 2023). Four species decreased in relative abundance: *Acutalibacter ornithocaccae, Klebsiella pneumoniae, Scatomorpha intestinigallinarum*, and *Faecousia sp000435995*, with *K. pneumoniae* decreasing by more than 2-fold. *Fifteen* participants showed a reduction, and 9 participants showed a reduction from detectable to essentially undetectable levels (Figure 3).

**Figure 1:**
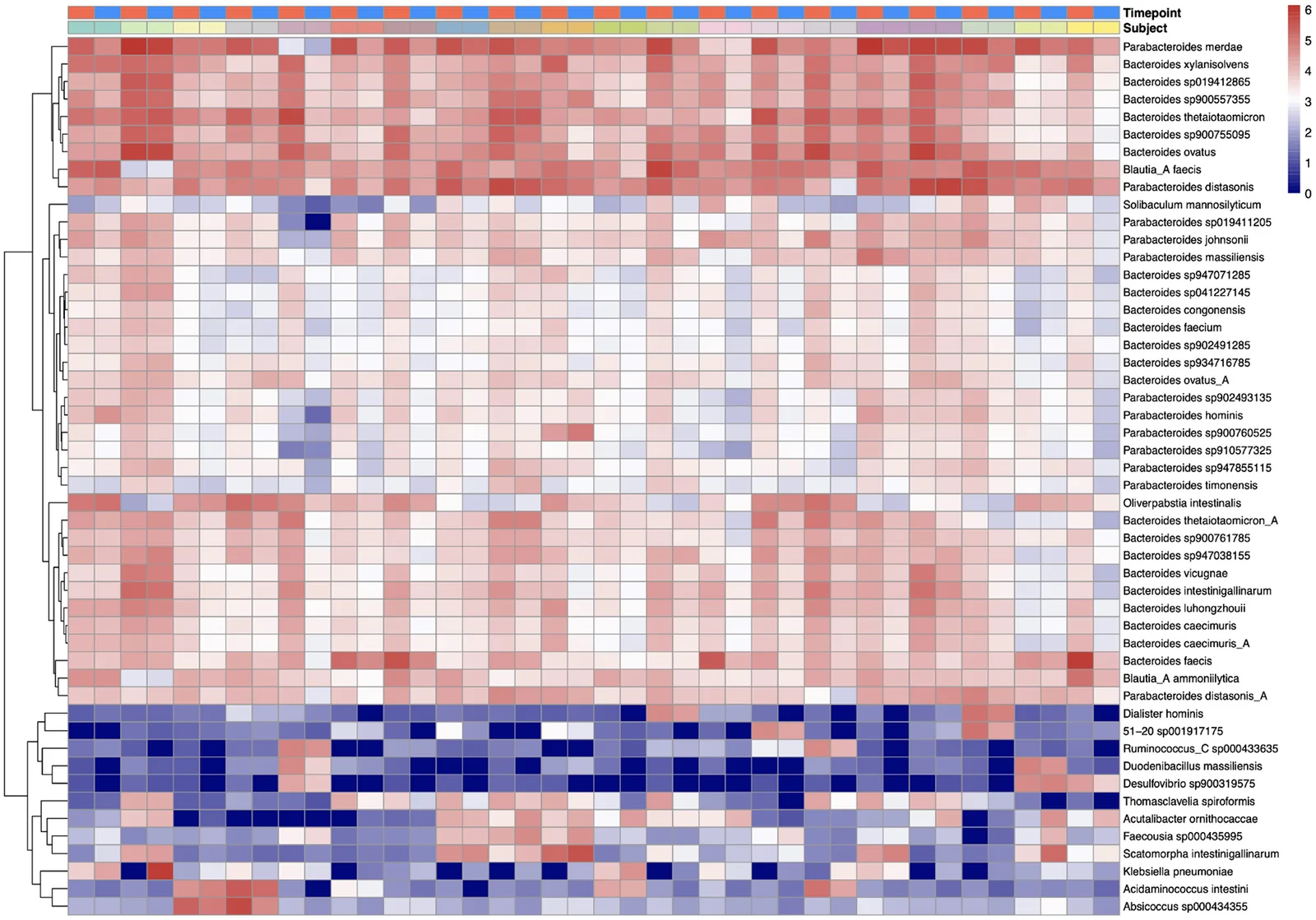
Heat map of the differential abundance of 50 species between baseline and the yeast mannan intervention in generally healthy adults aged 50 years or older. Timepoints: blue, baseline; orange, intervention.

**Figure 2:**
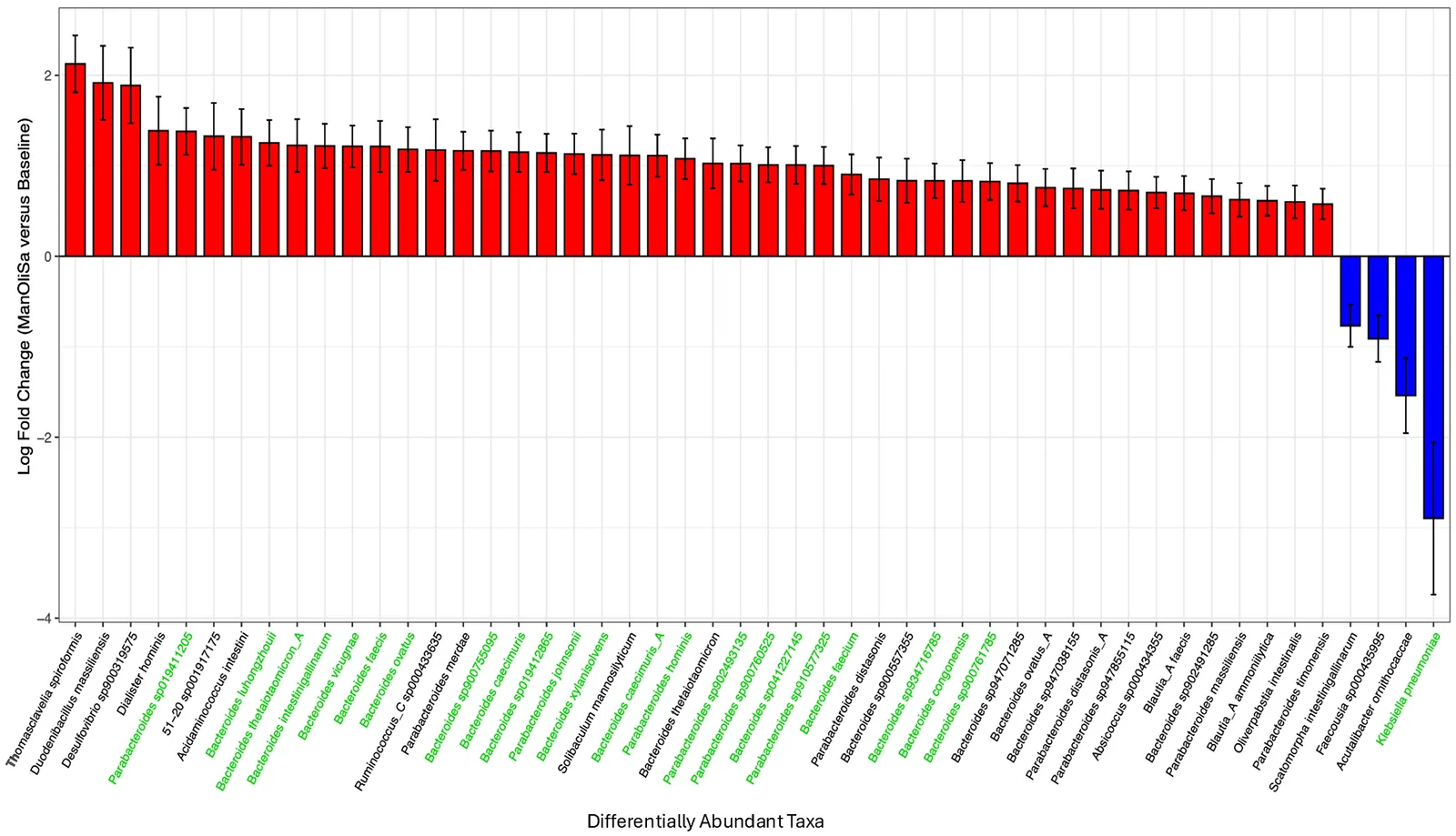
Differentially abundant taxa showing 50 significant taxa. Green indicates taxa that passed sensitivity analysis.

**Figure 3:**
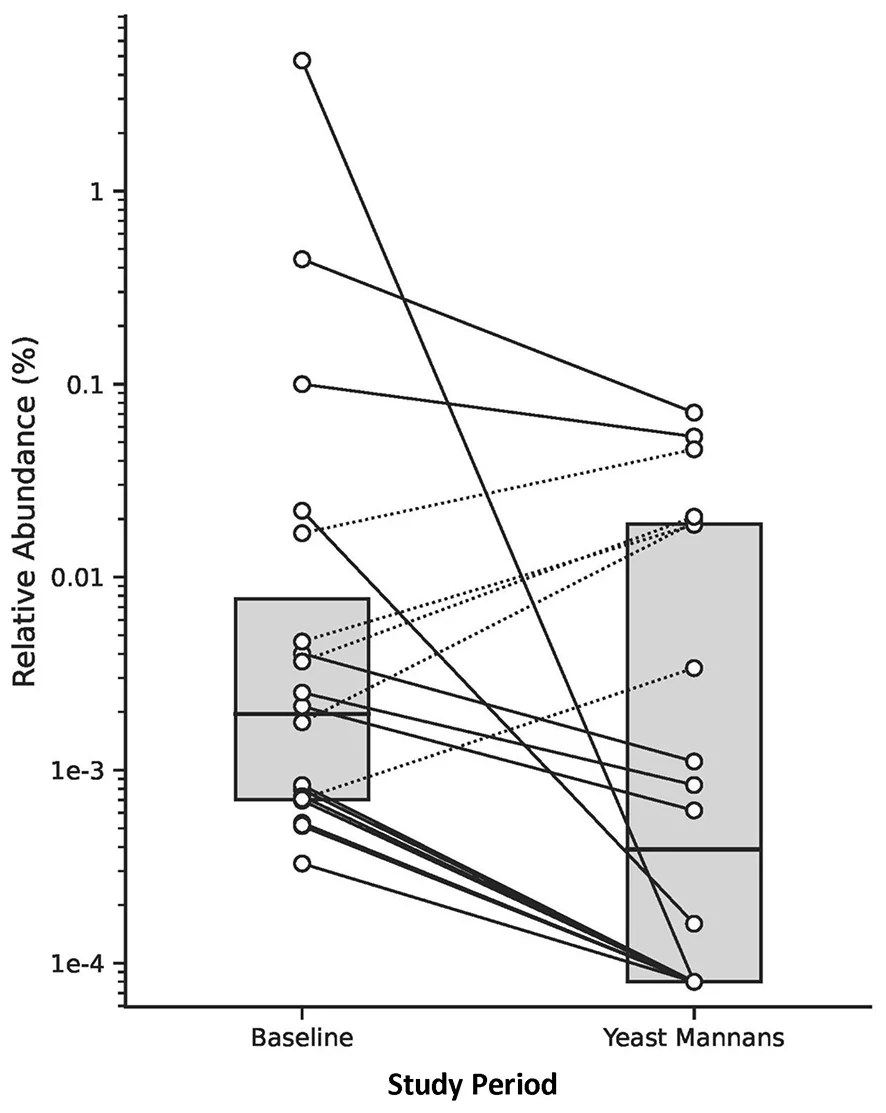
Relative abundance (percentage) of *Klebsiella pneumoniae* at baseline and following yeast mannan intervention (*n* = 20; *p* = 0.006). Gray boxes show the interquartile range; the center black line represents the median.

### 3.2 Metabolite associations with yeast mannan supplementation

Metabolite changes after yeast mannan administration were assessed between baseline and intervention in both urine and feces. There was a small set of consistent alterations in urine (*q* < 0.2). There was a distinct set of metabolites that differed in stool (*q* < 0.2). In urine, three metabolites significantly increased after yeast mannan administration: *N*-acetyl-alanine, alanine, and caproic acid. All three exceeded both the effect-size threshold (|coef| > 0.3) and the FDR threshold (*q* < 0.2), placing them in the upper-right quadrant of the volcano plot (Supplementary Figure S2). *N*-acetyl-alanine showed the strongest effect (largest coefficient and lowest q-value), followed by alanine and caproic acid. The coefficient plot (Figure 4) and paired heatmap (Figure 5) show a clear within-person upward shift for nearly all subjects. No urine metabolites showed a significant decrease after administration of yeast mannan.

**Figure 4:**
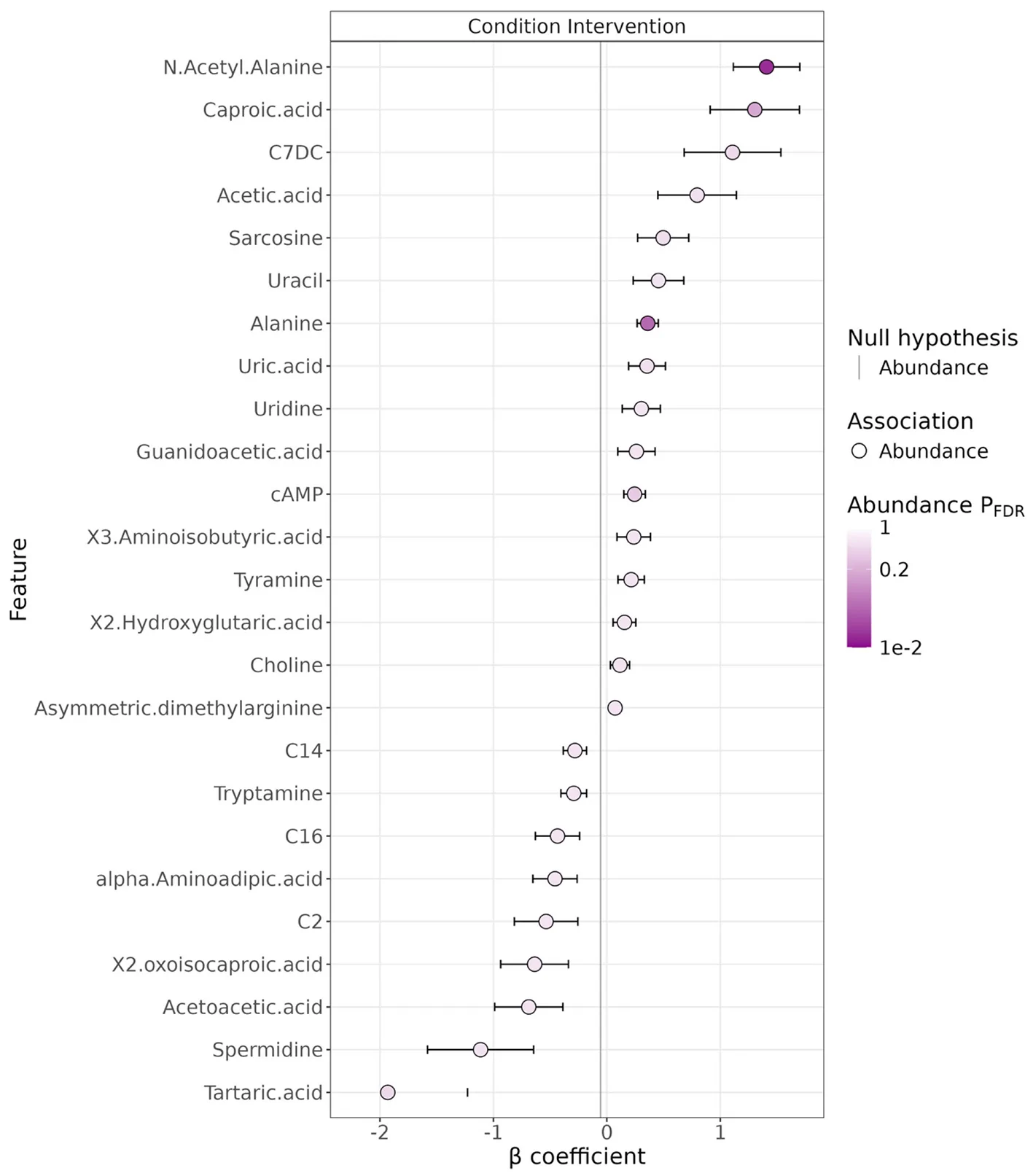
MaAsLin3 coefficient plot of 24-h urine metabolites at baseline and following yeast mannan supplementation.

**Figure 5:**
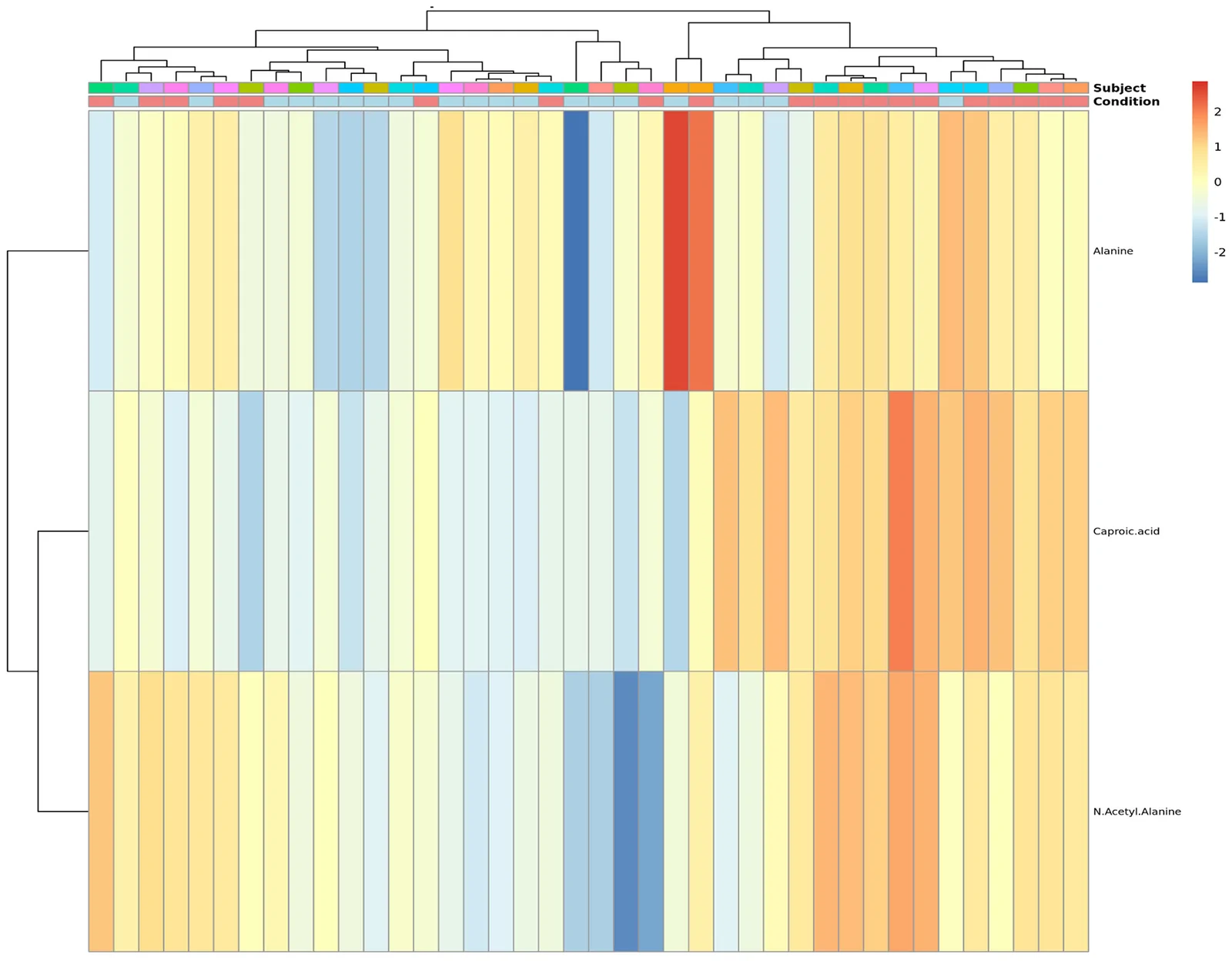
Paired heatmap of significantly associated 24-h urine metabolites (alanine, caproic acid, and *N*-acetylalanine) in response to yeast mannan intervention. Condition: light blue, baseline; orange, intervention.

In fecal samples, the dominant alterations were decreases in two lipid features. The acyl-alkyl phosphatidylcholines, PC ae C36:4 and PC ae C40:1, were lower after yeast mannan administration (Supplementary Figure S3). These ether-linked phosphatidylcholines also emerged as the strongest decreases in the coefficient plot (Figure 6) and displayed a paired downward shift across most subjects in the heatmap (Figure 7). No stool metabolites showed a significant increase after yeast mannan administration, although several features exhibited directional changes that did not survive false discovery rate correction.

**Figure 6:**
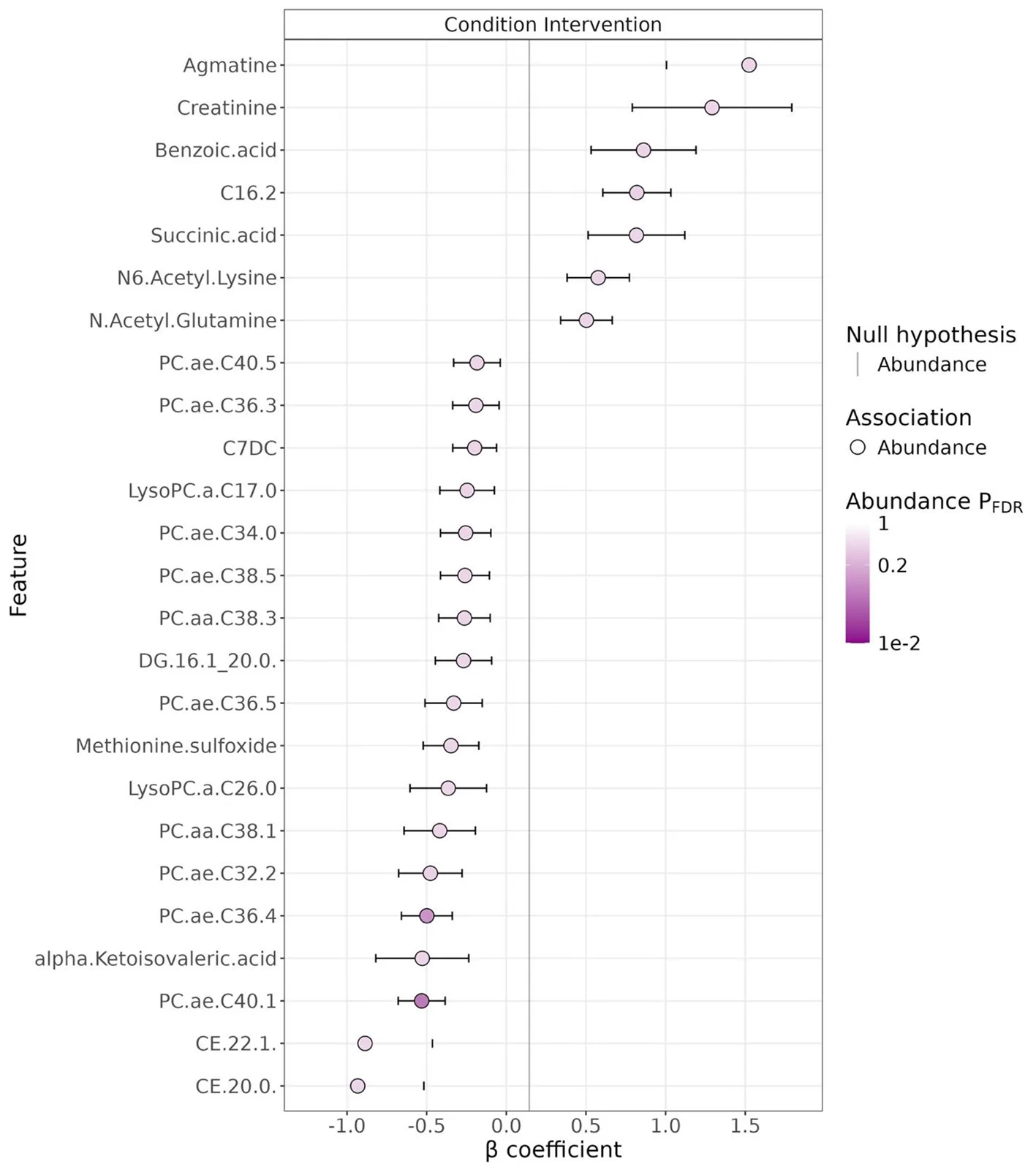
MaAsLin3 coefficient plot of fecal metabolites at baseline and following yeast mannan supplementation.

**Figure 7:**
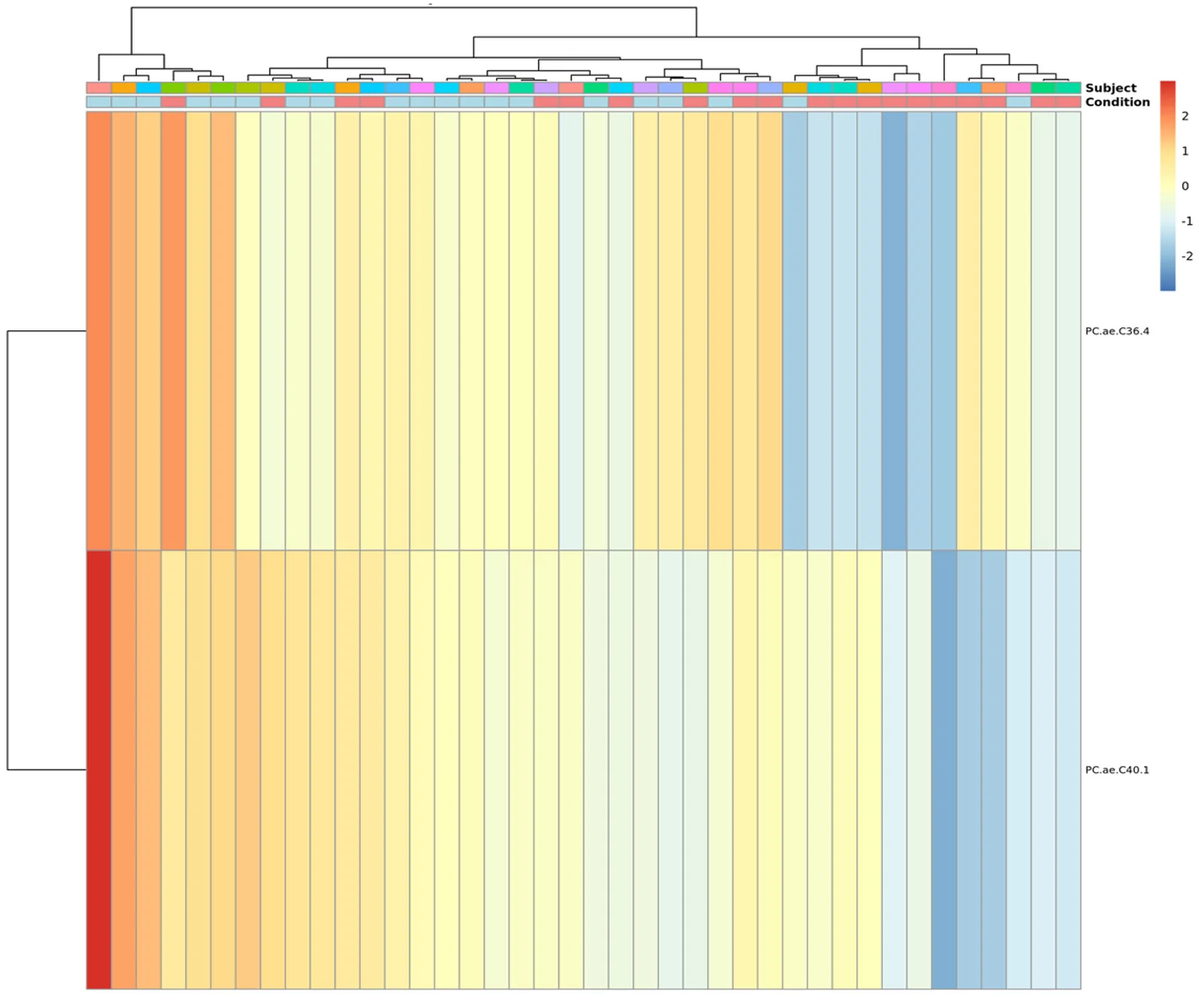
Paired heatmap of significantly associated fecal metabolites (PC ae C36:4 and PC ae C40:1) in response to yeast mannan intervention. Condition: light blue, baseline, orange, intervention.

The results of the pairwise sPLS integration of metabolome and microbiome show that at the genus level, correlations were widespread and primarily positive, forming a dense network centered around intervention-responsive urine metabolites such as taurine, carnosine, spermidine, hypoxanthine, putrescine, ethanolamine, and trans-4-hydroxyproline. Genera with the strongest co-variation (|*r*| > 0.6) included: *Roseburia_*B, *Oscillibacter, Veillonella*, UBA3402, CAG-510, *Mobilitalea, Sporaefaciens*, and *Kineothrix*. Amino acid–associated metabolites (e.g., taurine, carnosine, and putrescine) mapped to the largest and most connected network hubs, suggesting coordinated modulation of nitrogen-handling microbiota in relation to shifts in urine metabolism. Species-level relationships confirmed and refined genus patterns. Key species linked strongly to the metabolite cluster included: *Pilosibacter* spp., *Pullichristensenella stercoripullorum, Anaerotruncus massiliensis, Roseburia* sp934317215, and *Blautia_*A species. The most distinctive finding was that several species formed tight positive clusters around X7-methylguanine, ethanolamine, and C18 ceramides, indicating a coherent microbe–host axis influencing urine amino acid and lipid derivatives. Compared to urine, fecal microbe–metabolite associations were sparser and more selective, dominated by lipid-related metabolites such as p-cresol sulfate, phenylacetylglutamine, benzoic acid, C10/C12 acylcarnitines, isocitric acid, and shikimic acid. Key genera showing strong associations (|*r*| > 0.6) included: *Bifidobacterium, Faecalibacterium, Eubacterium*_Q, *Alectryocaccoccomibium*, UBA34302, and and *Colidextribacter*. Most associations were positive, suggesting that genera linked to saccharolytic fermentation may regulate fecal aromatic and acyl-chain metabolites. Species-level correlations highlighted an even more focused set of taxa: *Faecalibacterium prausnitzii* E, *Pilosibacter* sp900066785, *Enterocloster* sp900541315, *Bariatricus comes*, and *Pullichristensenella stercoripullorum*. These taxa showed the strongest relationships to *p*-cresol sulfate, phenylacetic acid, and phenylacetylglutamine, aromatic metabolites derived from host–microbial co-metabolism of phenylalanine. A particularly tight species cluster surrounded *p*-cresol sulfate, strongly implicating specific taxa from the Firmicutes lineage in its production or regulation.

## 4 Discussion

Results from the current analysis show that daily supplementation with a yeast mannan-rich ingredient was associated with a selective shift in fecal microbiota composition, characterized primarily by enrichment of *Bacteroides* and *Parabacteroides* species and a concurrent reduction in fecal *K. pneumoniae* relative abundance, including loss of detection in several participants. This response is consistent with the known capacity of selected Bacteroidota to utilize complex mannans and supports the concept that yeast mannan-rich substrates may act as targeted prebiotic ingredients for mannan-responsive commensals, potentially contributing to a gut microbial environment less favorable to opportunistic pathobionts.

Yeast mannans are highly branched cell-wall polysaccharides composed primarily of α-D-mannopyranose residues with an α-(1, 6)-mannan backbone and α-(1, 2)- and α-(1, 3)-linked mannose side chains, resulting in steric hindrance, which generally limits bacterial enzymatic degradation with the exception of some species within the phylum Bacteroidota (Cuskin et al., 2015; Hao et al., 2021). As hypothesized, yeast mannan supplementation enhanced the relative abundance of *Bacteroides* and *Parabacteroides* spp., consistent with previous 16S rRNA gene sequencing findings showing enrichment of *B. faecis, B. ovatus, P. merdae*, and *P. distasonis* (Moreno et al., 2025). An increase was also observed for *B. thetaiotaomicron*, consistent with Tanihiro et al. (2020), who examined supplementation with yeast mannans in healthy adult females. However, the present analysis also showed increases in 29 additional *Bacteroides* and *Parabacteroides* spp., as well as 12 other taxa. This skew toward increases in taxa is consistent with yeast mannan functioning primarily as a microbially accessible carbohydrate that can be metabolized by members of the gut community rather than acting as a broad-spectrum inhibitor. Both *Bacteroides* and *Parabacteroides* are well-known as dietary glycan generalists (Sonnenburg et al., 2010), with genomes enriched for polysaccharide utilization loci (PUL) that encode coordinated utilization of diverse complex carbohydrates (Grondin et al., 2017). A plausible mechanism is that the yeast mannan supplement provides substrates that can be captured by *Bacteroides* PUL systems specialized for mannan breakdown. For example, a dedicated mannan PUL has been characterized in *Bacteroides ovatus*, including GH26 β-mannanases that are upregulated during growth on mannan substrates (Bågenholm et al., 2017). Consistent with this model, independent *ex vivo*/*in vitro* fermentation studies report that yeast mannans selectively increase the relative abundance of *Bacteroides* (Oba et al., 2020b; Moreno et al., 2025).

The enrichment of *Bacteroides* and *Parabacteroides* may be beneficial because many Bacteroidota produce propionate (Fischbach and Sonnenburg, 2011; Reichardt et al., 2014). Beyond serving as an energy source for the host, propionate can influence mucosal and systemic physiology through G protein-coupled receptor signaling and other immunometabolic mechanisms (Koh et al., 2016). Recent mechanistic work has also directly tied *Bacteroides*-encoded propionate production genes to effects on intestinal homeostasis (Wang et al., 2024). In line with this potential homeostatic benefit, decreased relative abundance of *Bacteroides ovatus*, specifically, has been associated with dysbiosis in graft-vs.-host disease (GVHD), a life-threatening complication in patients following allogeneic hematopoietic stem cell transplantation (Hayase et al., 2024). In an animal model of GVHD, reintroduction of *Bacteroides ovatus* reduced dysbiosis, GVHD risk, and mortality, possibly by suppressing the mucus-degrading capabilities of *Bacteroides thetaiotaomicron* and *Akkermansia muciniphila* (Hayase et al., 2024). *B. thetaiotaomicron*, with its extensive glycan-degrading capacity and contribution to cross-feeding, has been shown to reduce inflammation in preclinical models of Crohn's disease (Delday et al., 2019) and slow the progression of colorectal cancer in murine models (Shi et al., 2026). However, the benefits of *B. thetaiotaomicron* are context-dependent, as this species can contribute to mucous degradation in the absence of microbial-available carbohydrate substrate in the diet (Desai et al., 2016). It is possible that yeast mannans, which provide a fermentable substrate for both *Bacteroides ovatus* and *Bacteroides thetaiotaomicron*, among others, may support gut barrier integrity by reducing competition for mucin glycoproteins and thus preventing degradation, with considerable potential for application in patient populations.

Yeast mannan supplementation increased the relative abundance of 12 *Parabacteroides* species. The consistent enrichment of multiple *Parabacteroides* species suggests that yeast mannan-rich substrates may support a broader commensal network than previously captured by 16S-based approaches. *Parabacteroides* are increasingly being explored as probiotic candidates due to their potential to mediate inflammatory and metabolic disorders (Sun et al., 2023; Chen et al., 2025). The relative abundance of *Parabacteroides*, as well as *Bacteroides*, has been shown to be reduced in obese adults, with an inverse relationship to body mass index, waist circumference, and cholesterol and triglyceride levels (Zeng et al., 2019). Additionally, selective enrichment of *Parabacteroides* with soluble corn fiber supplementation showed that greater increases in relative abundance were associated with cognitive benefits (Alvarado et al., 2026). Their limited shotgun metagenomics subsample of 3 responders compared with three non-responders identified *P. distasonis* as the primary species responsive to soluble corn fiber supplementation (Alvarado et al., 2026). The relative abundance of *P. distasonis* was similarly increased in the present study. Further research is needed to determine whether enriching *Parabacteroides* with yeast mannan supplementation offers metabolic and cognitive benefits.

The marked increase in *Thomasclavelia spiroformis* (formerly known as *Clostridium spiroforme*) after the yeast mannan intervention is consistent with yeast mannans altering the available carbohydrate landscape and downstream metabolite pools, potentially indirectly favoring *T. spiroformis* via cross-feeding, such as on mannose (Lawson et al., 2023) and community restructuring, given the lack of direct evidence that this species degrades yeast mannans. Other enhanced species include *Dialister hominis*, a succinate-utilizing bacterium, and *Acidaminococcus intestini*, an asaccharolytic bacterium that utilizes amino acids; the latter may have benefited from the provision of mannoproteins. Additionally, *Solibaculum mannosilyticum* was increased, which may be due to increased availability of mannose (Sakamoto et al., 2021). Two *Blautia spp*. were also increased*, B. faecis and B. ammoniilytica. Ex vivo* digestion/fermentation demonstrated that yeast mannans enriched *B. faecis* (Moreno et al., 2025) but not *B. ammoniilytica*. *Blautia* spp. may be secondary responders to yeast mannan fermentation, benefiting from metabolites or cross-feeding interactions rather than degrading mannan. Species-specific human health effects associated with higher fecal abundance of these taxa are lacking.

*Acutalibacter ornithocaccae, Klebsiella pneumoniae, Scatomorpha intestinigallinarum*, and *Faecousia sp000435995* decreased with the yeast mannan intervention. Because metagenomic profiles are inherently compositional (Gloor et al., 2017), this necessitates the use of tools such as Analysis of Compositions of Microbiomes with Bias Correction (ANCOM-BC2) designed to function on compositional data (Lin and Peddada, 2024). This means that the detected decreases in relative abundance are likely to reflect changes in absolute abundance; however, qPCR is needed to confirm these differences. Among these, the apparent reduction in *K. pneumoniae* is particularly notable given that it is a well-known opportunistic pathobiont. *K. pneumoniae* is one of the six ESKAPE pathogens (*Enterococcus faecium, Staphylococcus aureus, Klebsiella pneumoniae, Acinetobacter baumannii, Pseudomonas aeruginosa*, and *Enterobacter* spp.) exhibiting multidrug-resistant characteristics (Miller and Arias, 2024). *K. pneumoniae*, in particular, is thought to play a key role in the transfer of antimicrobial resistance (AMR) genes to other pathogens (Wyres and Holt, 2018). Additionally, *K. pneumoniae* is responsible for a significant burden of lower respiratory illness globally (Li et al., 2025) and neonatal sepsis (Belew et al., 2026), and its translocation leads to hepatic infections and contributes to hepatocellular carcinoma (Cheam and Gan, 2026; Wu et al., 2026). Furthermore, *K. pneumoniae* has been repeatedly linked to inflammatory bowel disease cohorts and experimental inflammatory phenotypes (Federici et al., 2022), as well as the infection burden in dialysis (Zhou et al., 2022; Zhang et al., 2024b) and solid organ transplant patients (Linares et al., 2010). In ICU patients, increasing intestinal abundance and dominance by *K. pneumoniae* were associated with greater illness severity and preceded bloodstream and abdominal infections, which were frequent among non-survivors (Mu et al., 2022). This risk of bloodborne infections has also been shown among long-term acute care hospital patients (Shimasaki et al., 2019). The yeast mannan-associated shift away from *K. pneumoniae*, to below-detection levels in 9 out of 20 study participants, may reflect improved colonization resistance or competitive suppression by carbohydrate-adapted commensals. Animal model evidence indicates that Bacteroidota mitigate *K. pneumoniae* colonization (Sequeira et al., 2020). Using a gnotobiotic approach in mice and a selection of human commensal bacteria, Furuichi et al. (2024) concluded that restricting nutrient availability may be the mechanism underlying improved resistance to *Klebsiella* colonization. Additional evidence suggests that the availability of complex carbohydrates mitigates colonization (Hecht et al., 2024). Interestingly, the carbohydrate form may determine the direction of the effect. Using an *in vitro* growth inhibition assay, mannooligosaccharides from coconut have been shown to inhibit the growth of *K. pneumoniae* (Asbury and Saville, 2025). Conversely, in a murine model, lactulose, a synthetic fermentable sugar, enhanced *K. pneumoniae* colonization (Hecht et al., 2024), and *in vitro* studies suggest that FOS also promotes *K. pneumoniae* colonization (Li et al., 2022). These examples raise a serious question regarding risk for hospital-acquired *K. pneumoniae* infections, given that lactulose is prescribed for constipation and encephalopathy, and FOS is added to many enteral formulas and nutritional supplements. The potential health benefit of yeast-mannan suppression of *K. pneumoniae*, particularly in patient populations at risk for nosocomial infections, requires exploration.

Beyond changes in the microbiome, yeast mannan supplementation altered the urinary and fecal metabolomes. Urinary excretion of *N*-acetylalanine increased; however, this may also reflect changes in kidney aminoacylase activity and amino acid metabolism (Lindner et al., 2008), so elevated levels cannot be strictly interpreted as increased bacterial production. However, previous *ex vivo* work using untargeted LC-MS demonstrated that yeast mannans stimulated the production of specific *N*-acetylated amino acids, namely *N*-acetylalanine and *N*-acetylphenylalanine. Additionally, using the same method, Van den Abbeele et al. (2025) have shown that yeast protein extract may be resistant to digestion, thereby providing a substrate for the microbiota, resulting in higher abundances of Bacillota and Bacteroidota and stimulating *N*-acetylation of amino acids. In the present study, the higher urinary *N*-acetylalanine levels may have been attributable to the supplement's mannoprotein component. Such stimulation of *N*-acetyl amino acids has been observed in response to human milk oligosaccharides, potentially contributing to early-life immune development and healthy gut barrier function (Martin et al., 2022). Whether there is a role for *N*-acetyl amino acids in the re-establishment of gut barrier function following disease or treatment-induced dysbiosis remains to be established, as does the potential beneficial role of yeast fractions. In addition, increased urinary excretion of caproic acid was observed with yeast mannan supplementation, a medium-chain fatty acid that is much less studied than the SCFAs, butyrate, propionate, and acetate. Caproic acid is not typically excreted in appreciable amounts; however, because it is found only in trace amounts in certain foods, increased urinary excretion may reflect increased production. The observed enrichment of *Bacteroides* and *Parabacteroides* in response to yeast mannan supplementation would be expected to favor propionate production. However, given the absorption, first-pass utilization, and metabolism efficiency of propionate (Boets et al., 2017), significant urinary levels were not expected.

Regarding fecal metabolites, Tanihiro et al. (2023) reported an increase in propionate with yeast mannans, and we did not. However, fecal concentrations do not necessarily reflect the rate of SCFA production (Sakata, 2019), so production may have increased, but absorption may also have increased. Fecal analysis did show changes in two ether-linked phosphatidylcholines, ae C36:4 and PC ae C40:1. There was an apparent, although not significant, increase in urinary excretion of choline and sarcosine. This could suggest targeted uptake of the phosphatidylcholines or microbial metabolism. Increased urinary excretion of caproic acid, acetic acid, and C7DC (pimeloylcarnitine), although the latter two were not significant, suggests that fatty acids from phosphatidylcholines may also be metabolized by human cells. No differences were observed in bile acids, which was somewhat unexpected, as phosphatidylcholines and bile acids are typically closely linked due to their co-secretion during lipid metabolism.

Finally, we sought to investigate which microbial genera and species show the strongest covariation with intervention-responsive metabolites, and whether these associations differ between urine and fecal samples. Across all four CSV-based analyses, a clear pattern emerged. For urine, many genera and species correlated with amino acid, polyamine, and nucleotide metabolites; the network structure was dense and highly connected. These findings suggest systemic metabolic signatures reflecting whole-body physiology. For feces, microbial associations concentrated around aromatic metabolites (*p*-cresol sulfate, phenylpropionylglycine), acylcarnitines, and organic acids, and the network structure was modular and lipid-focused, which perhaps indicates regulation of metabolites formed or transformed in the gut lumen. The pairwise sPLS analysis revealed that microbe–metabolite correlations are highly site-specific. Several microbial taxa identified (e.g., *Faecalibacterium, Roseburia*, and *Pullichristensenella*) also overlapped with sPLS-DA and MaAsLin3 findings, providing validation of their potential functional roles.

A limitation of this open-label pilot study is that plasma metabolomics were not measured, which limits the interpretation of whether yeast mannan supplementation produces systemic changes in the host through microbiota-derived and absorbed metabolites. Furthermore, there was no control group for comparison. Measuring plasma metabolites would provide insight into whether changes in stool frequency and gut microbiota composition were accompanied by alterations in circulating amino acid derivatives or other bioactive compounds. Such data may help identify metabolic pathways that warrant evaluation in larger randomized controlled trials.

Daily supplementation with a yeast mannan-rich ingredient was associated with selective modulation of the fecal microbiome, characterized by a selective enrichment of *Bacteroides* and *Parabacteroides* species and a concurrent reduction in fecal *K. pneumoniae* relative abundance, including loss of detection in several participants. Because *Bacteroides* and *Parabacteroides* are saccharolytic commensals with the capacity to utilize complex glycans, their enrichment may contribute to a microbial environment less favorable to opportunistic pathobionts. Future controlled studies, ideally incorporating absolute microbial quantification, will help determine whether the observed decrease in *K. pneumoniae* relative abundance reflects a true reduction in absolute abundance and whether these ecological shifts have physiological significance in selected populations where intestinal *K. pneumoniae* carriage may be of clinical concern.

## Data Availability

The data analyzed in this study are available in the NCBI repository under BioProject accession number PRJNA1549224 (https://www.ncbi.nlm.nih.gov/bioproject/PRJNA1549224).

## References

[B1] Alvarado D. A. Holthaus T. A. Martell S. Southey N. L. Atallah M. Sarma R. . (2026). Effects of soluble corn fiber consumption on executive functions and gut microbiota in middle to older age adults: a randomized controlled crossover trial. J. Nutr. 156:101473. doi: 10.1016/j.tjnut.2026.101473 41825740 PMC13197951

[B2] Asbury R. E. Saville B. A. (2025). Manno-oligosaccharides as a promising antimicrobial strategy: pathogen inhibition and synergistic effects with antibiotics. Front. Microbiol. 16:1529081. doi: 10.3389/fmicb.2025.1529081 40196030 PMC11973258

[B3] Bågenholm V. Reddy S. K. Bouraoui H. Morrill J. Kulcinskaja E. Bahr C. M. . (2017). Galactomannan catabolism conferred by a polysaccharide utilization locus of *Bacteroides ovatus*: enzyme synergy and crystal structure of a β-mannanase. J. Biol. Chem. 292, 229–243. doi: 10.1074/jbc.M116.746438 27872187 PMC5217682

[B4] Belew H. Tegegne B. A. Kindie Y. Tilahun A. Mihret M. Y. Teffera Z. H. . (2026). Bacterial neonatal sepsis, antimicrobial resistance, and risk factors among neonates in Ethiopia: a systematic review and meta-analysis. Ther. Adv. Infect. Dis. 13:20499361261451360. doi: 10.1177/20499361261451360 42222265 PMC13219930

[B5] Boets E. Gomand S. V. Deroover L. Preston T. Vermeulen K. De Preter V. . (2017). Systemic availability and metabolism of colonic-derived short-chain fatty acids in healthy subjects: a stable isotope study. J. Physiol. 595, 541–555. doi: 10.1113/JP272613 27510655 PMC5233652

[B6] Cheam G. Gan Y. H. (2026). Host interactions with hypervirulent *Klebsiella pneumoniae* along the gut-liver axis. Curr. Opin. Microbiol. 92:102764. doi: 10.1016/j.mib.2026.102764 42166807

[B7] Chen S. J. Wu Y. J. Chen C. C. Wu Y. W. Liou J. M. Wu M. S. . (2023). Plasma metabolites of aromatic amino acids associate with clinical severity and gut microbiota of Parkinson's disease. NPJ Parkinsons Dis. 9:165. doi: 10.1038/s41531-023-00612-y 38097625 PMC10721883

[B8] Chen Y. Jiang S. Wang H. Si M. Wu H. Liang X. . (2025). The probiotic *Parabacteroides johnsonii* ameliorates metabolic disorders through promoting BCAAs to BSCFAs conversion. Adv. Sci. 12:e02624. doi: 10.1002/advs.202502624 40772426 PMC12520484

[B9] Chumpitazi B. P. McMeans A. R. Vaughan A. Ali A. Orlando S. Elsaadi A. . (2018). Fructans exacerbate symptoms in a subset of children with irritable bowel syndrome. Clin. Gastroenterol. Hepatol. 16, 219.e211–225.e211. doi: 10.1016/j.cgh.2017.09.043 28970147 PMC5794652

[B10] Cummings J. H. Macfarlane G. T. (1991). The control and consequences of bacterial fermentation in the human colon. J. Appl. Bacteriol. 70, 443–459. doi: 10.1111/j.1365-2672.1991.tb02739.x 1938669

[B11] Cuskin F. Lowe E. C. Temple M. J. Zhu Y. Cameron E. Pudlo N. A. . (2015). Human gut Bacteroidetes can utilize yeast mannan through a selfish mechanism. Nature 517, 165–169. doi: 10.1038/nature13995 25567280 PMC4978465

[B12] Delday M. Mulder I. Logan E. T. Grant G. (2019). *Bacteroides thetaiotaomicron* ameliorates colon inflammation in preclinical models of Crohn's disease. Inflamm. Bowel Dis. 25, 85–96. doi: 10.1093/ibd/izy281 30215718 PMC6290787

[B13] Desai M. S. Seekatz A. M. Koropatkin N. M. Kamada N. Hickey C. A. Wolter M. . (2016). A dietary fiber-deprived gut microbiota degrades the colonic mucus barrier and enhances pathogen susceptibility. Cell 167, 1339.e1321–1353.e1321. doi: 10.1016/j.cell.2016.10.043 27863247 PMC5131798

[B14] Dou Y. Yu X. Luo Y. Chen B. Ma D. Zhu J. (2022). Effect of fructooligosaccharides supplementation on the gut microbiota in human: a systematic review and meta-analysis. Nutrients 14:3298. doi: 10.3390/nu14163298 36014803 PMC9413759

[B15] Federici S. Kredo-Russo S. Valdés-Mas R. Kviatcovsky D. Weinstock E. Matiuhin Y. . (2022). Targeted suppression of human IBD-associated gut microbiota commensals by phage consortia for treatment of intestinal inflammation. Cell 185, 2879.e2824–2898.e2824. doi: 10.1016/j.cell.2022.07.003 35931020

[B16] Fischbach M. A. Sonnenburg J. L. (2011). Eating for two: how metabolism establishes interspecies interactions in the gut. Cell Host Microbe 10, 336–347. doi: 10.1016/j.chom.2011.10.002 22018234 PMC3225337

[B17] Furuichi M. Kawaguchi T. Pust M. M. Yasuma-Mitobe K. Plichta D. R. Hasegawa N. . (2024). Commensal consortia decolonize Enterobacteriaceae via ecological control. Nature 633, 878–886. doi: 10.1038/s41586-024-07960-6 39294375 PMC11424487

[B18] Gibson G. R. Hutkins R. Sanders M. E. Prescott S. L. Reimer R. A. Salminen S. J. . (2017). Expert consensus document: the International Scientific Association for Probiotics and Prebiotics (ISAPP) consensus statement on the definition and scope of prebiotics. Nat. Rev. Gastroenterol. Hepatol. 14, 491–502. doi: 10.1038/nrgastro.2017.75 28611480

[B19] Gibson G. R. Roberfroid M. B. (1995). Dietary modulation of the human colonic microbiota: introducing the concept of prebiotics. J. Nutr. 125, 1401–1412. doi: 10.1093/jn/125.6.1401 7782892

[B20] Gloor G. B. Macklaim J. M. Pawlowsky-Glahn V. Egozcue J. J. (2017). Microbiome datasets are compositional: and this is not optional. Front. Microbiol. 8:2224. doi: 10.3389/fmicb.2017.02224 29187837 PMC5695134

[B21] Grondin J. M. Tamura K. Déjean G. Abbott D. W. Brumer H. (2017). Polysaccharide utilization loci: fueling microbial communities. J. Bacteriol. 199:e00860-16. doi: 10.1128/JB.00860-16 28138099 PMC5512228

[B22] Hao Z. Wang X. Yang H. Tu T. Zhang J. Luo H. . (2021). PUL-mediated plant cell wall polysaccharide utilization in the gut bacteroidetes. Int. J. Mol. Sci. 22:3077. doi: 10.3390/ijms22063077 33802923 PMC8002723

[B23] Harlacher E. Wollenhaupt J. Baaten C. Noels H. (2022). Impact of uremic toxins on endothelial dysfunction in chronic kidney disease: a systematic review. Int. J. Mol. Sci. 23:531. doi: 10.3390/ijms23010531 35008960 PMC8745705

[B24] Hayase E. Hayase T. Mukherjee A. Stinson S. C. Jamal M. A. Ortega M. R. . (2024). *Bacteroides ovatus* alleviates dysbiotic microbiota-induced graft-versus-host disease. Cell Host Microbe 32, 1621.e1626–1636.e1626. doi: 10.1016/j.chom.2024.08.004 39214085 PMC11441101

[B25] Hecht A. L. Harling L. C. Friedman E. S. Tanes C. Lee J. Firrman J. . (2024). Dietary carbohydrates regulate intestinal colonization and dissemination of *Klebsiella pneumoniae*. J. Clin. Invest. 134:e174726. doi: 10.1172/JCI174726 38512401 PMC11060737

[B26] Jensen L. G. Kjær M. Andersen J. R. (2025). Modification of the low FODMAP diet is feasible in the treatment of irritable bowel syndrome: a randomised crossover study. Gastroenterol. Res. Pract. 2025:4152978. doi: 10.1155/grp/4152978 41278484 PMC12634161

[B27] Kaneuchi C. Miyazato T. Shinjo T. Mitsuoka T. (1979). Taxonomic study of helically coiled, sporeforming anaerobes isolated from the intestines of humans and other animals: *Clostridium cocleatum* sp. nov. and *Clostridium spiroforme* sp. nov. Int. J. Syst. Evol. Microbiol. 29, 1–12. doi: 10.1099/00207713-29-1-1

[B28] Koh A. De Vadder F. Kovatcheva-Datchary P. Backhed F. (2016). From dietary fiber to host physiology: short-chain fatty acids as key bacterial metabolites. Cell 165, 1332–1345. doi: 10.1016/j.cell.2016.05.041 27259147

[B29] Lawson P. A. Saavedra Perez L. Sankaranarayanan K. (2023). Reclassification of *Clostridium cocleatum, Clostridium ramosum, Clostridium spiroforme* and *Clostridium saccharogumia* as *Thomasclavelia cocleata* gen. nov., comb. nov., *Thomasclavelia ramosa* comb. nov., gen. nov., *Thomasclavelia spiroformis* comb. nov. and *Thomasclavelia saccharogumia* comb. nov. Int. J. Syst. Evol. Microbiol. 73:005694. doi: 10.1099/ijsem.0.005694 36748617

[B30] Li H. Lane J. A. Chen J. Lu Z. Wang H. Dhital S. . (2022). *In vitro* fermentation of human milk oligosaccharides by individual *Bifidobacterium longum*-dominant infant fecal inocula. Carbohydr. Polym. 287:119322. doi: 10.1016/j.carbpol.2022.119322 35422288

[B31] Li J. Xu L. Zuo A. F. Xu P. Xu K. (2025). The global burden of *Klebsiella pneumoniae*-associated lower respiratory infection in 204 countries and territories, 1990-2021: findings from the global burden of disease study 2021. PLoS ONE 20:e0324151. doi: 10.1371/journal.pone.0324151 40388463 PMC12088015

[B32] Lin H. Peddada S. D. (2024). Multigroup analysis of compositions of microbiomes with covariate adjustments and repeated measures. Nat. Methods 21, 83–91. doi: 10.1038/s41592-023-02092-7 38158428 PMC10776411

[B33] Linares L. Cervera C. Hoyo I. Sanclemente G. Marco F. Cofán F. . (2010). *Klebsiella pneumoniae* infection in solid organ transplant recipients: epidemiology and antibiotic resistance. Transplant. Proc. 42, 2941–2943. doi: 10.1016/j.transproceed.2010.07.080 20970577

[B34] Lindner H. A. Täfler-Naumann M. Röhm K. H. (2008). *N*-acetylamino acid utilization by kidney aminoacylase-1. Biochimie 90, 773–780. doi: 10.1016/j.biochi.2007.12.006 18222180

[B35] Lu J. Breitwieser F. P. Thielen P. Salzberg S. L. (2017). Bracken: estimating species abundance in metagenomics data. PeerJ Comput. Sci. 3:e104. doi: 10.7717/peerj-cs.104 40271438 PMC12016282

[B36] Majid H. A. Cole J. Emery P. W. Whelan K. (2014). Additional oligofructose/inulin does not increase faecal bifidobacteria in critically ill patients receiving enteral nutrition: a randomised controlled trial. Clin. Nutr. 33, 966–972. doi: 10.1016/j.clnu.2013.11.008 24290345

[B37] Martin F. P. Tytgat H. L. P. Krogh Pedersen H. Moine D. Eklund A. C. Berger B. . (2022). Host-microbial co-metabolites modulated by human milk oligosaccharides relate to reduced risk of respiratory tract infections. Front. Nutr. 9:935711. doi: 10.3389/fnut.2022.935711 35990340 PMC9386273

[B38] McMurdie P. J. Holmes S. (2013). phyloseq: an R package for reproducible interactive analysis and graphics of microbiome census data. PLoS ONE 8:e61217. doi: 10.1371/journal.pone.0061217 23630581 PMC3632530

[B39] Miller W. R. Arias C. A. (2024). ESKAPE pathogens: antimicrobial resistance, epidemiology, clinical impact and therapeutics. Nat. Rev. Microbiol. 22, 598–616. doi: 10.1038/s41579-024-01054-w 38831030 PMC13147291

[B40] Moreno M. L. Abbeele P. V. D. Baudot A. Tompkins T. A. Taft D. H. Yao R. . (2025). Yeast mannans promote laxation and specifically modulate microbiota composition in older adults: an open-label pilot study. Nutr. Res. 136, 15–27. doi: 10.1016/j.nutres.2025.02.004 40117931

[B41] Mu S. Xiang H. Wang Y. Wei W. Long X. Han Y. . (2022). The pathogens of secondary infection in septic patients share a similar genotype to those that predominate in the gut. Crit. Care 26:68. doi: 10.1186/s13054-022-03943-z 35331299 PMC8944137

[B42] Mysonhimer A. R. Holscher H. D. (2022). Gastrointestinal effects and tolerance of nondigestible carbohydrate consumption. Adv. Nutr. 13, 2237–2276. doi: 10.1093/advances/nmac094 36041173 PMC9776669

[B43] Nemet I. Li X. S. Haghikia A. Li L. Wilcox J. Romano K. A. . (2023). Atlas of gut microbe-derived products from aromatic amino acids and risk of cardiovascular morbidity and mortality. Eur. Heart J. 44, 3085–3096. doi: 10.1093/eurheartj/ehad333 37342006 PMC10481777

[B44] Nickols W. A. Kuntz T. Shen J. Maharjan S. Mallick H. Franzosa E. A. . (2026). MaAsLin 3: refining and extending generalized multivariable linear models for meta-omic association discovery. Nat. Methods 23, 554–564. doi: 10.1038/s41592-025-02923-9 41540124 PMC12982127

[B45] Ntranos A. Park H. J. Wentling M. Tolstikov V. Amatruda M. Inbar B. . (2022). Bacterial neurotoxic metabolites in multiple sclerosis cerebrospinal fluid and plasma. Brain 145, 569–583. doi: 10.1093/brain/awab320 34894211 PMC10060700

[B46] Oba S. Sunagawa T. Tanihiro R. Awashima K. Sugiyama H. Odani T. . (2020a). Prebiotic effects of yeast mannan, which selectively promotes *Bacteroides thetaiotaomicron* and *Bacteroides ovatus* in a human colonic microbiota model. Sci. Rep. 10:17351. doi: 10.1038/s41598-020-74379-0 33060635 PMC7562712

[B47] Oba S. Washida K. Shimada Y. Sunagawa T. Tanihiro R. Sugiyama H. . (2020b). Yeast mannan increases *Bacteroides thetaiotaomicron* abundance and suppresses putrefactive compound production in *in vitro* fecal microbiota fermentation. Biosci. Biotechnol. Biochem. 84, 2174–2178. doi: 10.1080/09168451.2020.1784704 32597375

[B48] Parks D. H. Chuvochina M. Rinke C. Mussig A. J. Chaumeil P. A. Hugenholtz P. (2022). GTDB: an ongoing census of bacterial and archaeal diversity through a phylogenetically consistent, rank normalized and complete genome-based taxonomy. Nucleic Acids Res. 50, D785–D794. doi: 10.1093/nar/gkab776 34520557 PMC8728215

[B49] Reichardt N. Duncan S. H. Young P. Belenguer A. McWilliam Leitch C. Scott K. P. . (2014). Phylogenetic distribution of three pathways for propionate production within the human gut microbiota. ISME J. 8, 1323–1335. doi: 10.1038/ismej.2014.14 24553467 PMC4030238

[B50] Sakamoto M. Ikeyama N. Toyoda A. Murakami T. Mori H. Morohoshi S. . (2021). *Coprobacter secundus* subsp. similis subsp. nov. and *Solibaculum mannosilyticum* gen. nov., sp. nov., isolated from human feces. Microbiol. Immunol. 65, 245–256. doi: 10.1111/1348-0421.12886 33913539

[B51] Sakata T. (2019). Pitfalls in short-chain fatty acid research: a methodological review. Anim. Sci. J. 90, 3–13. doi: 10.1111/asj.13118 30370625 PMC6587520

[B52] Sequeira R. P. McDonald J. A. K. Marchesi J. R. Clarke T. B. (2020). Commensal *Bacteroidetes* protect against *Klebsiella pneumoniae* colonization and transmission through IL-36 signalling. Nat. Microbiol. 5, 304–313. doi: 10.1038/s41564-019-0640-1 31907407 PMC7610889

[B53] Serbanescu M. A. Wright M. C. Elebasy M. Shi P. Arnold J. W. Haines K. L. . (2025). Impact of fiber-containing enteral nutrition on microbial community dynamics in critically ill trauma patients: a pilot-randomized trial. BMC Med. 23:706. doi: 10.1186/s12916-025-04511-2 41462238 PMC12751656

[B54] Shi Z. Zou D. Hong S. Yu Y. Guo Z. (2026). *Bacteroides thetaiotaomicron* improves colorectal cancer prognosis by remodeling the immune microenvironment. J. Gastroenterol. Hepatol. 41, 20150–2058. doi: 10.1111/jgh.70412 42117299

[B55] Shimasaki T. Seekatz A. Bassis C. Rhee Y. Yelin R. D. Fogg L. . (2019). Increased relative abundance of *Klebsiella pneumoniae* carbapenemase-producing *Klebsiella pneumoniae* within the gut microbiota is associated with risk of bloodstream infection in long-term acute care hospital patients. Clin. Infect. Dis. 68, 2053–2059. doi: 10.1093/cid/ciy796 30239622 PMC6541703

[B56] Sonnenburg E. D. Zheng H. Joglekar P. Higginbottom S. K. Firbank S. J. Bolam D. N. . (2010). Specificity of polysaccharide use in intestinal bacteroides species determines diet-induced microbiota alterations. Cell 141, 1241–1252. doi: 10.1016/j.cell.2010.05.005 20603004 PMC2900928

[B57] Sun H. Guo Y. Wang H. Yin A. Hu J. Yuan T. . (2023). Gut commensal *Parabacteroides distasonis* alleviates inflammatory arthritis. Gut 72, 1664–1677. doi: 10.1136/gutjnl-2022-327756 36604114

[B58] Tanihiro R. Sakano K. Oba S. Nakamura C. Ohki K. Hirota T. . (2020). Effects of yeast mannan which promotes beneficial bacteroides on the intestinal environment and skin condition: a randomized, double-blind, placebo-controlled study. Nutrients 12:3673. doi: 10.3390/nu12123673 33260560 PMC7761098

[B59] Tanihiro R. Yuki M. Sasai M. Haseda A. Kagami-Katsuyama H. Hirota T. . (2023). Effects of prebiotic yeast mannan on gut health and sleep quality in healthy adults: a randomized, double-blind, placebo-controlled study. Nutrients 16:141. doi: 10.3390/nu16010141 38201970 PMC10780920

[B60] Van den Abbeele P. Vu L. D. Poppe J. van Hengel I. A. J. Baudot A. Zhang Y. . (2025). Yeast protein modulates metabolites derived from the human gut microbiota of older male adults *ex vivo* to strengthen gut barrier function and reduce inflammation. Front. Microbiol. 16:1697734. doi: 10.3389/fmicb.2025.1697734 41568030 PMC12816187

[B61] van Lanen A. S. de Bree A. Greyling A. (2021). Efficacy of a low-FODMAP diet in adult irritable bowel syndrome: a systematic review and meta-analysis. Eur. J. Nutr. 60, 3505–3522. doi: 10.1007/s00394-020-02473-0 33585949 PMC8354978

[B62] Vandeputte D. Falony G. Vieira-Silva S. Wang J. Sailer M. Theis S. . (2017). Prebiotic inulin-type fructans induce specific changes in the human gut microbiota. Gut 66, 1968–1974. doi: 10.1136/gutjnl-2016-313271 28213610 PMC5739857

[B63] Wang X. Cai Z. Wang Q. Wu C. Sun Y. Wang Z. . (2024). Bacteroides methylmalonyl-CoA mutase produces propionate that promotes intestinal goblet cell differentiation and homeostasis. Cell Host Microbe 32, 63.e67–78.e67. doi: 10.1016/j.chom.2023.11.005 38056459

[B64] Wei Q. (2025). Yeast mannan: a comprehensive functional analysis from structural characteristics to biological activity. Carbohydr. Res. 558:109715. doi: 10.1016/j.carres.2025.109715 41145032

[B65] Wood D. E. Lu J. Langmead B. (2019). Improved metagenomic analysis with Kraken 2. Genome Biol. 20:257. doi: 10.1186/s13059-019-1891-0 31779668 PMC6883579

[B66] Wu M. Zhang Y. Yu J. (2026). How the gut microbiome affects the immunotherapy response in hepatocellular carcinoma. Cancer Biol. Med. 23:20250761. doi: 10.20892/j.issn.2095-3941.2025.0761 41968757 PMC13244310

[B67] Wyres K. L. Holt K. E. (2018). *Klebsiella pneumoniae* as a key trafficker of drug resistance genes from environmental to clinically important bacteria. Curr. Opin. Microbiol. 45, 131–139. doi: 10.1016/j.mib.2018.04.004 29723841

[B68] Xu J. Bjursell M. K. Himrod J. Deng S. Carmichael L. K. Chiang H. C. . (2003). A genomic view of the human-*Bacteroides thetaiotaomicron* symbiosis. Science 299, 2074–2076. doi: 10.1126/science.1080029 12663928

[B69] Zeng Q. Li D. He Y. Li Y. Yang Z. Zhao X. . (2019). Discrepant gut microbiota markers for the classification of obesity-related metabolic abnormalities. Sci. Rep. 9:13424. doi: 10.1038/s41598-019-49462-w 31530820 PMC6748942

[B70] Zhang L. Zheng J. Johnson M. Mandal R. Cruz M. Martínez-Huélamo M. . (2024a). A comprehensive LC-MS metabolomics assay for quantitative analysis of serum and plasma. Metabolites 14:622. doi: 10.3390/metabo14110622 39590858 PMC11596266

[B71] Zhang L. Zhang H. Su S. Jia Y. Liang C. Fang Y. . (2024b). Risk factor assessment and microbiome analysis in peritoneal dialysis-related peritonitis reveal etiological characteristics. Front. Immunol. 15:1443468. doi: 10.3389/fimmu.2024.1443468 39611142 PMC11602453

[B72] Zhou S. Ren G. Liu Y. Liu X. Zhang L. Xu S. . (2022). Challenge of evolving *Klebsiella pneumoniae* infection in patients on hemodialysis: from the classic strain to the carbapenem-resistant hypervirulent one. Int. J. Med. Sci. 19, 416–424. doi: 10.7150/ijms.69577 35370471 PMC8964319

