# Supplementary material for: Nutritional intervention trial of yeast mannans derived from baker's yeast enriches Bacteroides and Parabacteroides with concurrent reduction of Klebsiella pneumoniae
Source: Front Microbiol. 2026 Oct 30;17:1958679. doi: 10.3389/fmicb.2026.1958679 (PMC13760527; doi:10.3389/fmicb.2026.1958679)
Supplement: Supplementary file 1 [file Data_Sheet_1.docx]

Nutritional intervention trial of yeast mannans derived from baker’s yeast enriches *Bacteroides* and *Parabacteroides* with concurrent reduction of *Klebsiella pneumoniae*

**Supplementary Materials**

**Supplemental Methods: Bray-Curtis distance**

Bray-Curtis distance was used to measure beta diversity. PERMANOVA (adonis2) with restricted permutations was used to test differences in beta-diversity between baseline and yeast mannan intervention.

**Fig. S1**. Beta diversity between the baseline and yeast mannan as displayed in non-metric multi-dimensional Scaling (NMDS). No significant difference was noted.

**Fig. S2.** MaAsLin3 volcano plot showing change in 24-h urine metabolomics between baseline and yeast mannan supplementation.

**Fig. S3**. MaAsLin3 volcano plot of differences in fecal metabolomics comparing baseline to yeast mannan supplementation

**Supplementary Methods: Standard and Multilevel PCA**

To characterize global shifts in fecal and urine metabolomes between baseline and intervention, unsupervised principal component analysis (PCA) was performed on the paired datasets. Standard PCA, which does not account for paired study designs, showed no clear group separation in either fecal or urine data (Fig. S3). Baseline and intervention samples were extensively intermingled, and the principal components primarily captured inter-individual variability rather than intervention-driven changes. In contrast, applying multilevel PCA, which explicitly removes between-subject variation and isolates within-subject changes, revealed a markedly different structure (Fig. S3). For both fecal and urine metabolomes, multilevel PCA increased the proportion of variance attributed to the first component (fecal PC1: 33.9% → 38.3%; urine PC1: 19.8% → 17.3%) and partially separated baseline from intervention scores. This indicates that intervention effects were subtle and largely masked by strong subject-specific metabolomic signatures.

Next, subject-level metabolic trajectories were visualized by linking each individual's baseline and intervention PCA scores. In the multilevel PCA, arrows showed consistent directional movement for several subjects in both matrices, whereas standard PCA trajectories were disordered and dominated by baseline inter-subject differences (Fig. S4). This confirmed that adjusting for subject identity is essential to resolve biologically relevant intervention effects. Variance explained plots further underscored the benefit of multilevel decomposition (Fig. S4). In both fecal and urine analyses, multilevel PCA redistributed variance from higher components into PC1 and PC2, increasing the proportion of variance attributable to within-subject differences—precisely the dimension along which intervention effects should manifest. This reshaping of the variance structure justified the use of paired multilevel models for subsequent supervised analyses.

Finally, to examine paired differences directly, PC1 and PC2 deltas (Intervention – Baseline) were computed for each subject (Fig. S5). Fecal PC1 changes showed a right-skewed distribution with several subjects shifting strongly in the positive direction, consistent with metabolite increases following intervention. Urine PC1 responses were more evenly distributed with modest effect sizes. Cross-matrix correlations of PC1 differences were weak (r ≈ 0.10), indicating that fecal and urine metabolomes responded partially independently and supporting the hypothesis that combined multi-omics approaches would be necessary to capture coordinated metabolic signatures.

Cohen’s drm effect sizes (paired standardized differences) for standard PCA components were uniformly small in both matrices. However, multilevel PCA increased effect sizes for fecal PC2 (drm = 0.38) and reduced noise in urine PC1/PC2, suggesting that within-subject centering enhances sensitivity to detect subtle metabolic shifts (Fig. S6). Multivariate Hotelling’s T² tests provided converging evidence. For fecal multilevel PC1–PC2 space, the T² statistic exceeded the significance threshold (T² > 40, p < 0.05; Fig. S6), whereas urine changes did not reach significance. These results demonstrate that the fecal metabolome exhibits a more coordinated response to intervention than the urine metabolome.

Loadings from standard and multilevel PCA highlighted distinct sets of metabolites as major contributors to PC1 variation (Fig. S7). In fecal samples, amino acid–related metabolites (leucine, valine, tyrosine, serine, N-acetylated amino acids) dominated both PCA modes, although their relative contributions shifted after multilevel adjustment. Urine PC1 loading profiles were driven by organic acids (methylmalonic acid, isocitric acid, malonic acid) and methylated metabolites (asymmetric dimethylarginine), suggesting that intervention effects were distributed across different metabolic pathways across matrices.

Plotting correlations between loadings and intervention condition further revealed metabolites that consistently increased or decreased post-intervention (Fig. S8), establishing a preliminary signature that can be refined through supervised approaches.

**Fig. S4.** Standard principal component analysis (PCA) versus Multilevel PCA of fecal and urinary metabolomes at baseline and following a yeast mannan intervention.

**Fig. S5:** Paired trajectory analysis of fecal and urine metabolomes in response to yeast mannans demonstrates heterogeneous but directional within-subject shifts.

**Fig. S6.** Principal component difference histograms of fecal and urine metabolome.

**Fig. S7.** Effect sizes and Hotelling’s T² significance for standard and multilevel PCA components.

**Fig. S8.** PCA Loadings from standard and multilevel PCA of fecal and urine metabolome in response to yeast mannans.

**Fig. S9.** Condition-focused loading plots of fecal and urine metabolome in response to yeast mannans.
